# SUSSOL—Using Artificial Intelligence for Greener Solvent Selection and Substitution

**DOI:** 10.3390/molecules25133037

**Published:** 2020-07-03

**Authors:** Hannes Sels, Herwig De Smet, Jeroen Geuens

**Affiliations:** Centre of Expertise on Sustainable Chemistry, Department of Sciences and Technology, Karel de Grote Applied University of Arts and Sciences, 2660 Antwerp, Belgium; herwig.desmet@kdg.be

**Keywords:** solvent selection, solvent substitution, sustainable solvents, software, artificial intelligence, self-organizing map, SH&E

## Abstract

Solvents come in many shapes and types. Looking for solvents for a specific application can be hard, and looking for green alternatives for currently used nonbenign solvents can be even harder. We describe a new methodology for solvent selection and substitution, by applying Artificial Intelligence (AI) software to cluster a database of solvents based on their physical properties. The solvents are processed by a neural network, the Self-organizing Map of Kohonen, which results in a 2D map of clusters. The resulting clusters are validated both chemically and statistically and are presented in user-friendly visualizations by the SUSSOL (Sustainable Solvents Selection and Substitution Software) software. The software helps the user in exploring the solvent space and in generating and evaluating a list of possible alternatives for a specific solvent. The alternatives are ranked based on their safety, health, and environment scores. Cases are discussed to demonstrate the possibilities of our approach and to show that it can help in the search for more sustainable and greener solvents. The SUSSOL software makes intuitive sense and in most case studies, the software confirms the findings in literature, thus providing a sound platform for selecting the most sustainable solvent candidate.

## 1. Introduction

Solvents are frequently used as diluents in chemical production processes and products like paint, cleaning agents, glue, and ink. The global solvent market is about 20 million tons a year [1]. The paints, cleaning, and pharmaceutical industries represent the main sectors with more than 60% of the total consumption of solvents [2]. In 2017, paints and coating (46%) and pharmaceutical industry (9%) combined solvent usage accounted for 55% in Europe [3]. Most classical solvents are nonrenewable, nonbiodegradable, flammable, or toxic and therefore, exhibit various problems in manipulation, recycling, and waste treatment [4].

The European regulation concerning the “Registration, Evaluation, Authorisation and Restriction of Chemicals (REACH)” was adopted to improve the protection of human health and the environment from the risks that can be posed by chemicals [5]. Solvents such as cyclohexane, benzene, toluene, chloroform and dichloromethane can be found in the REACH restriction list [6]. Chemicals that are candidates for future bans or restrictions are listed in the “candidate list of substances of very high concern (SVHC) for authorization” [7]. The candidate list for SVHC contains some solvents including nitrobenzene, *o*-toluidine, *N*-methylacetamide, *N*,*N*-dimethylformamide, furan, formamide, *N*,*N*-dimethylacetamide, *N*-methylpyrrolidone, 2-(m)ethoxyethanol, trichloroethylene, 1,2,3-trichloropropane, and 2,4-dinitrotoluene.

ChemSec is an independent nonprofit organization that advocates for substitution of toxic chemicals to safer alternatives [8]. Among other initiatives, ChemSec developed the “SIN-list” (Substitute It Now-list). The SIN-list consists of chemicals that ChemSec has identified as fulfilling the criteria for SVHC as defined by the EU chemicals regulation REACH [9]. Currently, the SIN list contains 991 chemicals, which makes it a stricter list than the SVHC list. Recently (November 2019) ChemSec’s SIN-list was updated with 47 chemicals [10], including a handful of solvents (e.g., 1,1,1-trichloroethane, trifluoroacetic acid, 1,4-dioxane, acetaldehyde, and 1,2-dihydroxybenzene).

This legislative pressure is one of the main drivers that steers industry towards greener alternatives for toxic solvents [11]. Furthermore, costs of disposing toxic solvents or recycling by distillation can have a significant impact on production costs. In the pharmaceutical industry, solvents typically contribute for 56% to the materials used to manufacture an active pharmaceutical ingredient (API) [12]. In a typical alkyd resin formulation, solvent content ranges between 15% and 55% (for heavy duty applications) by mass [13,14,15,16]. In addition, it has been estimated that solvents contribute 50% of the post-treatment greenhouse gas emissions of pharmaceutical manufacturing [17].

Additionally, increased consumer awareness can be a driver for manufacturers to look for alternatives in their consumer products [11]. The LIFE AskREACH project [18] has recently conducted surveys of more than 14,000 citizens in 14 European countries, targeting consumer awareness about SVHCs in consumer products. The survey shows that Europeans are highly concerned about the presence of problematic chemicals in products. In 9 (out of 14) countries, 70% of the respondents was highly or extremely concerned about the possibility that everyday products may have problematic substances that can be harmful to human health and the environment. At the same time, the study also reveals that EU citizens are not well informed [19]. According to REACH article 33 [20], upon request, consumers have the right to receive information from the suppliers about the presence of any SVHC in a product, its subassemblies, or its packaging above a threshold of 0.1% (weight/weight). The AskREACH project encourages consumers to make use of their “SVHC right to know” through the use of an app [21]. Initiatives like the AskREACH project, the ECHA ‘Chemicals in our life’ webpage [22], ChemSec, and other national and local consumer organizations can point companies to their corporate responsibility and drive manufacturers to more benign solutions.

In the past, pharmaceutical industry has already put great efforts into the substitution of toxic or hazardous solvents. Said efforts were mainly focused on safety, health, and environmental impact with limited consideration of the physical properties from the solvents in dispute. Several pharmaceutical companies developed their own substitution guides in the form of a table which lists preferred solvents and solvents that are to be avoided. The CHEM21 consortium did a great effort reviewing [23] the GSK [17,24,25], AstraZeneca [26,27], ACS GCI-PR [28], Sanofi [29], Rowan University [30], and ETH Zürich [31,32] solvent guides. Eventually this resulted into creating a consensus across previously mentioned guides and developing a solvent guide [33] that is widely supported, at least in the pharmaceutical industry. Although pharmaceutical industry has put the most effort in solvent awareness, it should be noted that major solvent use is situated in paints and coatings industry [3].

Byrne et al. [34] published an extensive review on previously developed solvent guides. Similarities and discrepancies of existing solvent guides [17,24,25,26,27,28,29,30,31,32,33,35] are discussed and the authors conclude that “there is no need for more general-purpose solvent selection guides of the familiar format because they are no longer providing any significant advancement in this field.” Moreover, “the solvent selection guide format has reached its potential.” However, we believe that there is a need for a data-driven, automated solvent selection/substitution guide. Such a guide would allow nonexpert users, not familiar with the field of solvent selection, to look for greener solvents and create more sustainable products and processes. Moreover, the software we introduce is based on Artificial Intelligence (AI) algorithms, resulting in a self-learning, extensible application for the future.

State-of-the-art AI supported quantitative structure property relationship (QSPR) modeling makes use of deep neural networks (DNN). Among others, AI supported QSPRs have been used for the prediction of octanol–water partition coefficients [36], solvation free energies [37], gas chromatographic retention indices [38]. and critical properties [39]. Often SMILES strings are used as the input for the DNN model [36,37,38,39]. Such an approach proves useful in the screening and development of green solvents with respect to unconventional and novel compounds. Interestingly, the methodology can be inversed (iQSPR) to generate new structures based on a set of molecular properties [40,41]. Deep neural network supported QSPR modeling exploits the concept of automatic feature extraction. Based on the input vector (SMILES or other), dominant structural features of the given compound are extracted by the neural network [37]. This technique requires a complex deep learning architecture. In contrast, the software presented in this publication makes use of a dataset of experimental physical properties, thus the features are known beforehand. Consequently, a complex deep learning architecture would be unsubstantiated for such use. Instead, a “shallow” neural network is used in this work (see Section 3.4.1).

Sustainable solvent alternatives are available but the search for the best solution is often time consuming, labor intensive, and requires specific knowledge. Moreover, nonexpert scientists often first draw up a list of potential solvents based on their own experience (“What did I use in the past?”) and from a pragmatic point of view (“What do we have in stock?”) and then make a choice based on (unsystematic) trial and error. In the past decade, considerable efforts were made especially in the pharmaceutical industry. However, for SME’s and small formulating companies where chemistry is not the core business and R&D culture is lacking, the search for a new and more sustainable solvent is not obvious. As Jessop concluded in his 2011 publication “Academic research in the area of green solvents is currently not focused on the applications that make the greatest contribution to the environmental impact of solvents” [42].

To enable a more efficient, objective, and purposeful selection of solvents, a user-friendly software tool—SUSSOL (Sustainable Solvents Selection and Substitution Software)—was developed, using AI. The aim of SUSSOL is to support companies in the search for sustainable and viable alternatives for nonbenign solvents currently used in their products and processes. Our software tries to bridge the gap between academic research and applicability in industry. By providing a flexible tool, companies can use it according to their own needs.

Previously, two software-based solvent guides have been published. Both use the physical properties of solvents to attain a data-driven and objective solvent selection. Scientists from AstraZeneca [27] developed a solvent selection tool based on a dataset of 272 solvents, characterized by 30 physical properties. This solvent dataset is visualized in a three-dimensional map and can be explored by the user. The tool allows to interactively select solvents based on the principal component analysis (PCA) of the solvents’ physical properties. Solvents which are close to each other on the map have similar physical and chemical properties, whereas solvents at a distance are significantly different [43]. In addition to the PCA scores, other data including the physical properties, functional groups, and environmental data has been included to aid in the rational selection of solvents. Meanwhile, the tool is validated by the ACS GCI Pharmaceutical Roundtable and publicly available on the ACS website [43]. The tool offers excellent insight; however, it is a bit slow as a web-based tool. It is clearly focused on the pharmaceutical industry and engineering. Most important, it does not offer flexibility to the user to add new solvents or additional (meta) data.

The second software-based guide is published by Tobiszewski et al. [44]. The authors use Ward’s hierarchical clustering analysis [45] to analyze a set of 151 solvents. The similarity between the solvents in the multidimensional space can be determined by the Euclidean distance between the solvents in the dataset. Three clusters (groups) of solvents with similar properties are determined. Tobiszewski et al. describe a group of “rather nonpolar and volatile compounds,” a group of “nonpolar and sparingly volatile solvents,” and a cluster with “polar solvents.” Within each cluster, the solvents are ranked by means of a multiple-criteria decision analysis technique (TOPSIS) [46,47]. Each solvent is assigned a calculated score between 1 and 0. Score 1 being the ideal solution, value 0 being the nonideal solution. The scores are benchmarked against the scores from the Pfizer [35], GCI-PR [28], GlaxoSmithKline [17,24,25], AstraZeneca [26], Sanofi [29], and the CHEM21 survey [23] solvent guides. The authors conclude that the ranking of solvents within each cluster generally agrees with other solvent selection guides [44]. However, the ranking of solvents with TOPSIS suffers from a lack of data. Hence, the authors split their ranking into different levels of confidence which does not benefit to the usability of the solvent guide. The guide is more of an academic exercise, than a ready-to-use tool.

Byrne et al. [34] state that this approach has reiterated that certain types of solvent have inherently undesirable characteristics and therefore, solvent selection on a direct ‘like-for-like’ substitution basis is restrictive. They also conclude that “Relying only on the existing catalogue of largely conventional solvents, it is not possible to have a green solvent substitute readily available for every application.”

Academic efforts to develop benign and biobased solvents should continue, but these new solvents should find their way to SME’s and small nonresearch-intensive companies. An SME with a small product portfolio does not have the means nor the knowledge to start the search for a better solvent. Software-aided tools could be of great help, and our tool attempts to enable this search for better solvents. Companies that are not aware of the relevant physical solvent properties characterizing their products and processes will find a great deal of help in the described software.

We think of a solvent selection/substitution tool as an interactive data-centered catalogue, both with conventional and neoteric solvents, that guides the user towards the best possible alternative. This process should be effortless and transparent. Providing flexibility to the users to work with their own dataset, add new solvents, and add company-specific or confidential data will certainly facilitate the use of this tool in an industrial environment. Furthermore, solvent producers and distributors of green and neoteric solvents can use the tools as a benchmark to promote their solvents as an alternative for conventional nonbenign solvents.

The presented SUSSOL software uses a solvent dataset in the form of a .csv file. The software [48] can be operated in two modes, the selection mode (Section 3.3 Solvent Selection) and the substitution mode (Section 3.4 Solvent Substitution). The solvent selection mode consists of a Multidimensional Scaling (MDS) plot of all solvents in the dataset. In the solvent substitution mode solvents are clustered into groups (clusters) of similar solvents, based on their physical properties. For this cluster analysis, the Self-organizing Map (SOM) from Kohonen [49,50] is used. The clusters are ranked on a two-dimensional grid where the distance between two clusters is a measure of similarity. The user is able to specify the solvent he wants to replace. After cluster analysis, a “candidate list” with similar solvents is generated. After selecting the most sustainable candidates, laboratory testing can take place.

The software and underlying principles and algorithms are described in detail in Section 3. Materials and Methods. We recommend to read this section first before proceeding with Section 2. Results and Discussion.

## 2. Results and Discussion

To validate the MDS plot and the clustering approach via the SOM algorithm a number of case studies were selected from literature. In addition, a case study from a real-world industrial application was investigated using the SUSSOL software.

### 2.1. Solvent Selection

Multidimensional scaling (MDS) [51,52] is a method that represents measures of similarity (or dissimilarity) among objects of which multiple (>2) properties can be quantified. The graphical display of the correlations provided by MDS enables the data analyst to literally “look” at the data and to explore their structure visually [51]. Our dataset contains 22 physical properties (Section 3.2 Dataset), hence the solvents are objects in a 22-dimensional space. MDS is used to reduce the number of dimensions to 2 for better visualization on a chart. Moreover, the distances between the solvents are also logged to file for further processing in other software packages.

**Figure 1:**
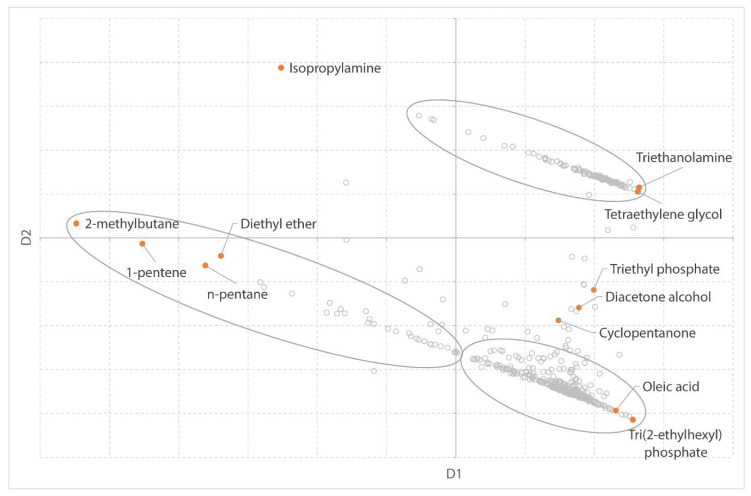
Multidimensional scaling (MDS) plot of all 500 solvents based on their 22 physical properties. Dimension 1 (D1) on x-axis and dimension 2 (D2) on y-axis. Three main groups of solvents are highlighted.

The dataset, containing 22 physical properties, is visualized in 2D using MDS in Figure 1. For the sake of visualization, the solvents are plotted in a chart, rather than displaying a screenshot of the MDS plot in the SUSSOL software.

To evaluate whether the MDS analysis accurately represents the proximity values as distances on the map, the normalized stress value (σ_n_) is used. This value is the proportion of variation of the dissimilarities not accounted for by the distances in the MDS map [51]. $1-\sigma_{n}$ is the fitted proportion, thus a coefficient of determination [51]. The normalized stress for the MDS plot (Figure 1) is 0.027, whereas the coefficient of determination is 0.97. The success in representing the actual data on a 2D map is also reflected by the two dimensions (D1 and D2), which account for 84.5% of the variance.

**Figure 2:**
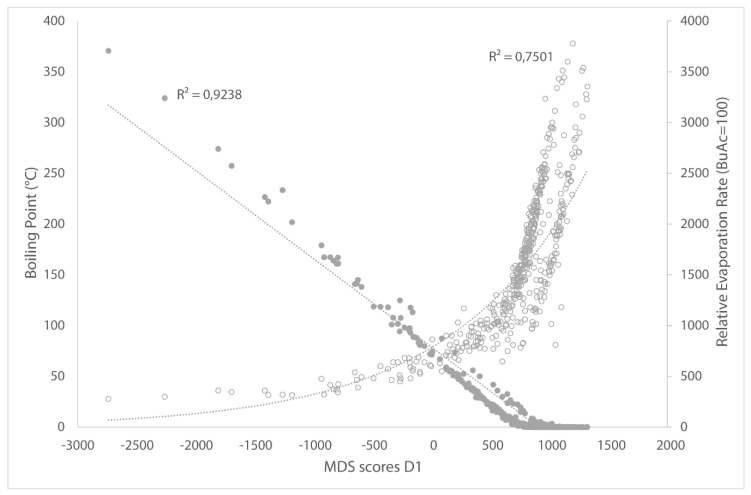
Correlations between D1 from MDS plot and boiling point and relative evaporation rate. Filled dots represent relative evaporation rate, whereas empty dots show boiling point.

The axes on the MDS plot are dimensionless. However, a trend can be observed in the datapoints of the solvents, which makes it possible to assign physical properties to the axes. The properties were assigned to the axes by visually observing the solvents in the MDS map and searching for correlations between D1 and D2 and the relevant physical properties. This helps the user to make some intuitive sense of the dimensionless axes but it is intended for that use only. Naming the axes with the correlated physical properties would be wrong since there is no sole contributor to every axis. Dimension 1 (the x-axis) in the MDS plot is a measure of volatility. From left to right in the visualization a gradient from low boiling solvents (2-methylbutane, 1-pentene, *n*-pentane, diethyl ether, and isopropylamine have boiling points lower than 36 °C) to high boiling solvents (triethanolamine, tetraethylene glycol, oleic acid, and tri(2-ethylhexyl)phosphate have a boiling point higher than 325 °C) is observed. This is confirmed by the strong correlations between the MDS scores from D1 and the boiling point and relative evaporation rate (Figure 2).

**Figure 3:**
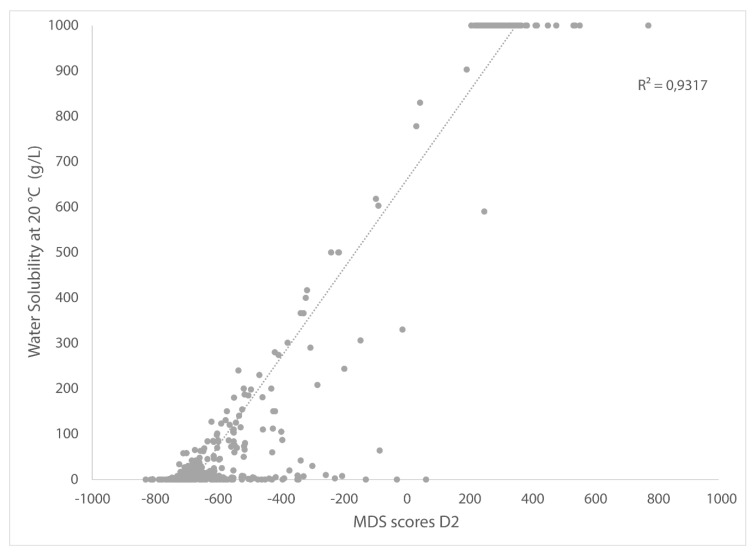
Correlation between D2 from MDS plot and water solubility.

It is less obvious to assign a physical property (or multiple properties) to Dimension 2 (the y-axis). A correlation between the y-scores from the MDS and water solubility can be found (Figure 3). However, the y-axis is also linked to other physical properties which are mainly linked to solvent polarity, as discussed in the following case studies. The graphic shows horizontal lines at 0 and 1000 g/L because these are the minimum and maximum values in the dataset for water solubility. Alkanes, alkenes, aromatic solvents, and most of the halogenated solvents are plotted on the lower half of Figure 1, whereas amines, short-chain alcohols, and dipolar aprotic solvents are depicted in the upper half.

We can roughly identify three groups of solvents in the visualization (Figure 1). A group of relatively high boiling, polar compounds, a group of low boiling, slightly water-soluble solvents, and a group of high boiling, hydrophobic solvents. In between the polar solvents and the group of high boiling, hydrophobic solvents, one can find solvents such as diacetone alcohol, triethyl phosphate, and cyclopentanone.

The three groups of solvents correspond to the three clusters found by Tobiszewski et al. [44]. In their work, the authors define the clusters as “rather nonpolar and volatile compounds,” “nonpolar and sparingly volatile solvents,” and “polar solvents.”

**Figure 4:**
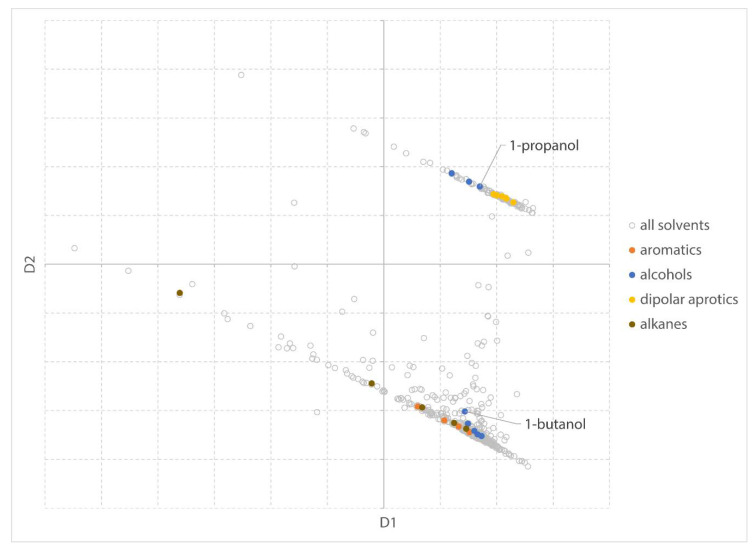
MDS plot of all solvents present in the dataset. Dimension 1 (D1) on x-axis and dimension 2 (D2) on y-axis. Small subsets of aromatic solvents, alcohols, dipolar aprotic solvents, and alkanes are highlighted.

In the MDS visualization, solvents that are “similar” are plotted closer together in the 2D solvent space as can be seen in Figure 4 where a group of typical dipolar aprotic solvents (DMF, DMAc, NMP, and DMSO) is highlighted in the MDS plot. Homologous series (e.g., alcohols, alkanes, and aromatic compounds with increasing alkyl chain) can be identified as a trend in the MDS plot (Figure 4).

The relative influence of a functional group decreases with increasing chain length and molecular size. Homologous series, therefore, are not always following a straight line in the MDS visualization, as can be seen in the homologous series from alcohols. Due to a drastic increase in boiling point and decrease in water solubility, vapor pressure, and relative evaporation rate, the series of alcohols shows a sudden jump from 1-propanol to 1-butanol.

#### 2.1.1. Solvent Effect in Keto–Enol Tautomerism

**Figure 5:**
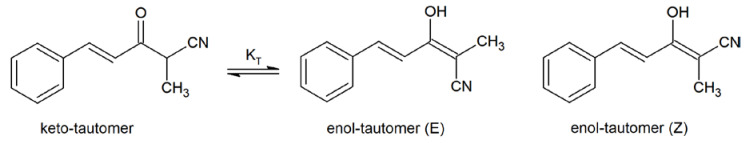
Keto–enol equilibrium for 2-methyl-5-phenyl-3-oxo-4-pentenenitrile.

**Figure 6:**
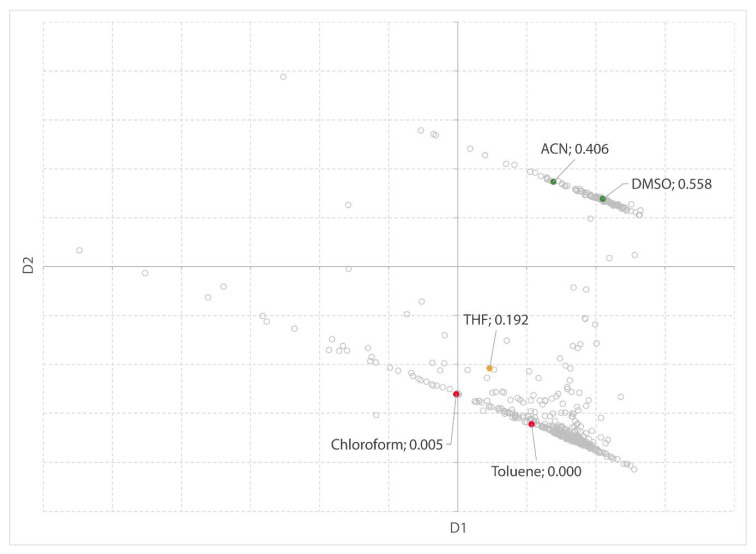
MDS plot with solvents highlighted in green (=high K_T_), amber (=medium K_T_), and red (=low K_T_). Solvent names and K_T_ values are shown as labels in the chart.

The keto–enol equilibrium of a compound in various solvents can be used as a case to test the MDS plot. The keto-enol tautomerization constant for 2-methyl-5-phenyl-3-oxo-4-pentenenitrile (Figure 5) is visualized through a color scale in the MDS plot. A top-down trend can be observed in the MDS chart (Figure 6), which indicates the dependency of the amount of enol-tautomers on solvent polarity. Giussi et al. [53] show that π* is the more important term in a linear solvation energy relationship (LSER) suggesting that the solute–solvent dipole–dipole interactions occur preferably. This makes sense as the (calculated) dipole moment of the keto-tautomer is lower than for the enol-tautomers (*E*- and *Z*- isomers), i.e., 3.34 D and 4.26 D and 4.99 D, respectively [46].

#### 2.1.2. Reaction Solvents for the Synthesis of Butyl Butanoate

**Figure 7:**
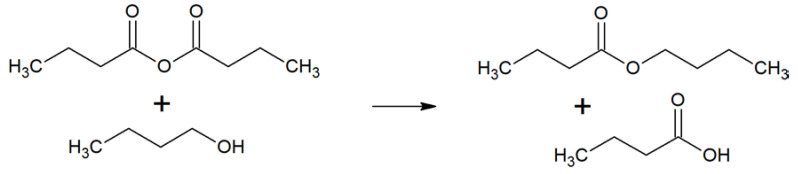
The synthesis of butyl butanoate from butanoic anhydride and 1-butanol.

**Figure 8:**
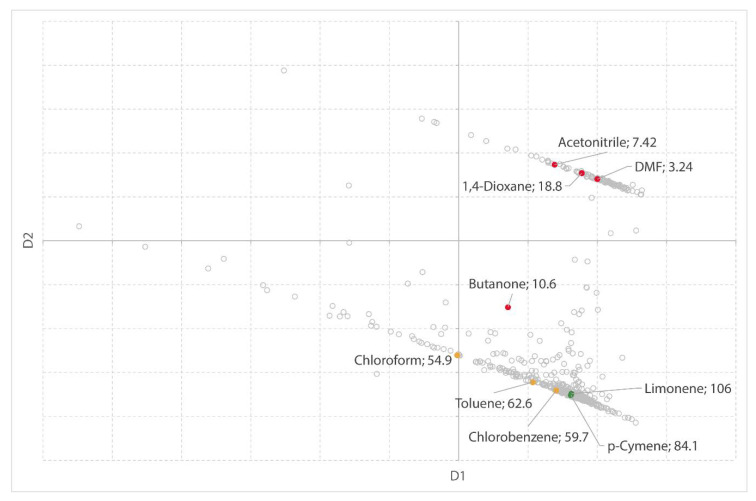
MDS plot with solvents highlighted in green (= high k_2_), amber (= medium k_2_), and red (= low k_2_). Solvent names and k_2_ values (10^−6^ dm³ mol^−1^ s^−1^) are shown as labels in the chart.

Next, the conversion of butanoic anhydride and 1-butanol into butyl butanoate (Figure 7) is used as a test for the MDS visualization. The second-order reaction rate constants in various solvents are visualized on the MDS chart (Figure 8). Again, the trend is apparent, although the rate constant for 1,4-dioxane seems too high to fit the trend. Clark et al. [54] make use of an LSER to demonstrate that β (hydrogen bond accepting ability) and δ²_H_ (the square of the Hildebrand solubility parameter) combined account for the rate of the esterification reaction. The remaining parameters (α and π*) were found not to be statistically significant.

From this data, it becomes clear that not only polarity-polarizability (π*) but also hydrogen-bond basicity (β) contributes to the y-axis in the MDS visualization.

#### 2.1.3. Liquid–Liquid Extraction of Furfural

**Figure 9:**
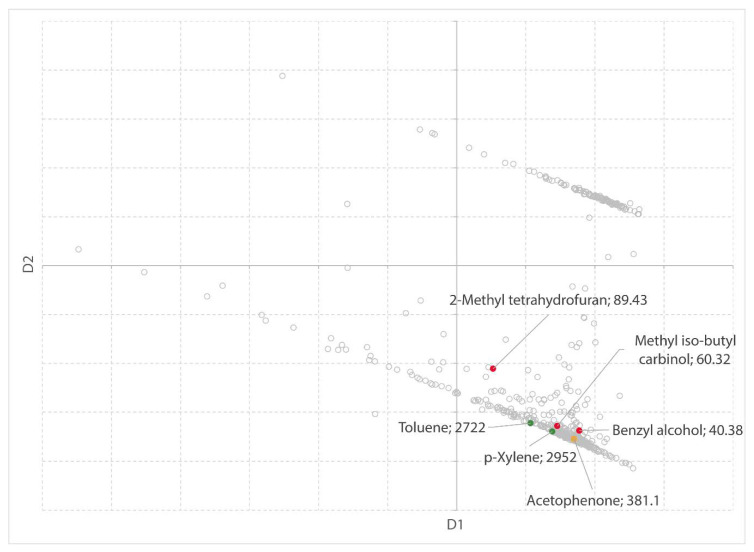
MDS plot with solvents highlighted in green (= high S), amber (= medium S), and red (= low S). Solvent names and S values are shown as labels in the chart.

Not in all test cases, a clear trend can be observed in the MDS plot. In the following example, solvents used for liquid–liquid extraction from furfural from an aquatic medium [55] are depicted in the MDS plot (Figure 9). The experimental separation factor is used as a measure for extraction efficiency.

(1) $$S=\frac{x_{2\beta }/x_{1\beta }}{x_{1\alpha }/x_{1\alpha }}$$

x_2α_ and x_1α_ are the mole fractions of solute and diluent in the aqueous phase, respectively. x_2β_ and x_1β_ are the mole fractions of solute and diluent, respectively, in the organic phase.

The trend in this case is not very obvious, however, a region of interest can be observed which may indicate solvents worth testing in laboratory experiments. It should be noted that Xin et al. [55] did not consider the removal of the extraction solvent. Concentrations of solvents and solutes in the organic and aqueous phase were determined using gas chromatography. In our dataset, multiple parameters are linked to the solvent’s evaporation behavior (boiling point, vapor pressure, relative evaporation rate, and Antoine constants), and therefore, it has a great impact on the MDS map.

### 2.2. Solvent Substitution

Three cases are analyzed with the SUSSOL software. In the first case study, the replacement of NMP is analyzed using the full dataset. In the second case, the substitution of toluene in a real-world application is studied using a subset of the full dataset. The third case study is performed from a solvent producer or distributor point of view. In the latter case, tetramethyl oxolane (TMO) was selected for a benchmark analysis against common organic solvents as well as other “green,” neoteric, and biobased solvents.

#### 2.2.1. Replacement of NMP

The fluorination via a S_N_Ar reaction is typically promoted by conventional dipolar aprotic solvents [56]. As such, the reaction is a good case to study possible substitutes for NMP.

The full dataset with 500 solvents and 21 physical properties was clustered on a map of 15 × 15 (225 centroids), and 2500 runs were executed on an Intel^®^ Core™ i7-8850H CPU @ 2.60 GHz. The total computation time was approximately 11 min: clustering 3 min and statistical analysis 8 min.

**Figure 10:**
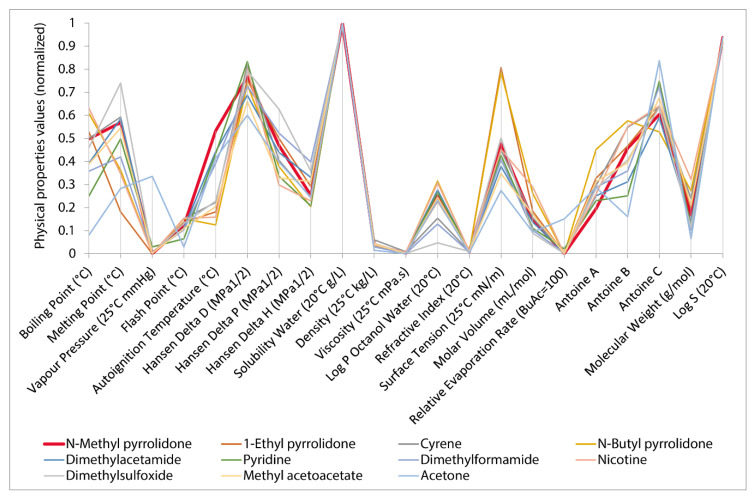
Normalized visualization of 10 solvents from candidate list with smallest Hansen distance towards NMP. Solvents are represented by a polyline.

**Figure 11:**
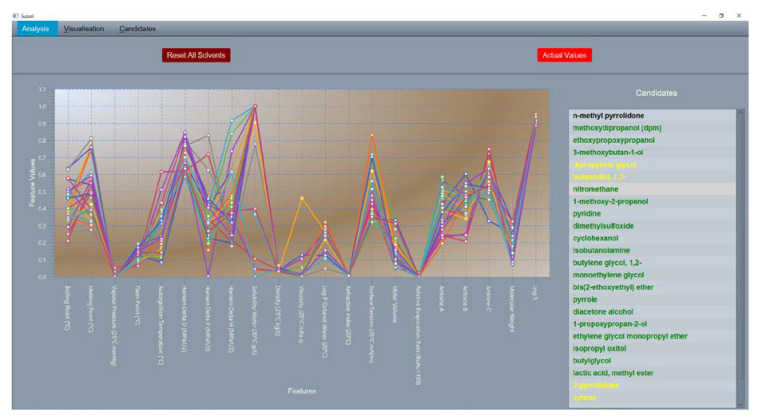
Visualization of candidates list in SUSSOL software. View can be switched between normalized and actual values. The candidate list in the screenshot is not complete. In the software, the scroll bar can be used to view the complete list. Protic solvents are still present in the list in the screenshot.

The clustering of the dataset and the following statistical analysis yielded 84 candidates. Since an aprotic solvent was needed and no physical parameter quantifying that property was present in the dataset, protic solvents were removed manually from the candidates. After removal of the protic solvents, 20 solvents remained as a possible substitute for NMP in the fluorination reaction. The top 10 candidate replacement solvents sorted on Hansen distance to NMP are visualized in a graph with a normalized y-axis, where 0 represents the lowest value in the 500 solvent dataset and 1, represents the highest value (Figure 10). Each solvent is represented as a polyline. More overlap between the polylines signifies similarity between solvents. The same visualization is used in the SUSSOL software (Figure 11). This allows the user to graphically asses the replacement candidates. In this case, there is quite a bit of variation for some properties. For certain properties like surface tension, the variation is not a problem, but other properties are key to the solvent’s performance. In this case, the solvent’s polarity and water solubility have a high impact on the performance of the solvent in the reaction. The solvents 1-formylpiperidine, furfural, 2,5-hexanedione, and diketene are poorly water soluble and cause the relative standard deviation for water solubility to be 40% (Figure 11). By giving this property more weight in the clustering process, a better output, tailored to the application under consideration, could be achieved. This feature has been provided in the SUSSOL software. The relative standard deviation drops to 4% after applying more weight to water solubility (Figure 10). The aforementioned solvents disappear from the candidates list.

In the SUSSOL software, the solvent names are color coded in accordance with the CHEM21 solvent guide. If the candidate list is sorted according to the CHEM21 health score (Table 1), DMSO appears to be the healthiest option for the replacement of NMP, followed by Cyrene and *N*-butyl pyrrolidone. Cyrene has the advantage that it is based on renewable feedstock. The authors of the study successfully tested 5 solvents in the S_N_Ar fluorination: DMSO, DMF, sulfolane, NMP, and Cyrene. All of the solvents are present in the candidates list, with exception of sulfolane, which is not present in the dataset.

#### 2.2.2. Replacement of Toluene

The Belgian company Soudal [57] produces silicone, caulks, polyurethane-foams, and adhesives. One of their neoprene rubber-based contact adhesives contains 75–80% of solvent. In the past, toluene was used to dissolve the neoprene rubber. Nowadays, due to legislation, toluene is banned from all formulations for the do-it-yourself market. To replace toluene, a mixture of 5 solvents (alkanes and others) was used to produce the adhesive. However, the composition of the mixture changes as the solvents evaporate with different evaporation rates, which may lead to (partial) insolubility of the neoprene rubber. Depending on environmental conditions, this may lead to an inhomogeneous film and/or affect the adhesion in a negative way. The most straightforward solution to overcome this problem would be a one-on-one replacement of toluene. Requirements are the complete dissolution of the polymer/resin mixture, a comparable evaporation profile, and a cost-efficient solution.

A subset of the 500 solvents dataset was created, using the Hansen distance to toluene as a measure. All solvents with a distance less than 4 MPa^1/2^ to toluene were included in the subset. The correct radius of the neoprene rubber was unknown, however, the smallest reported radius in HSPiP is 6.2. A rule of thumb by Seymour [58] describes two nonpolar compounds are miscible if they are within a distance of approximately 4 MPa^1/2^. To assure the complete solubility of the rubber in the solvent candidates, a distance of 4 MPa^1/2^ was adopted. The resulting set of 42 solvents was clustered on a 7 × 7 map. In total, 500 runs were executed. The generated solvent replacement candidates were ranked according to the health, environmental, and safety score (Table 2). The two first solvents in the ranking were selected for further laboratory testing. *p*-Cymene can be synthesized from renewable feedstock and is previously presented as a possible substitute for toluene [54]. Dipentene, limonene, and α-pinene are promising candidates because of their renewable origin. Nonetheless, these solvents were not tested because of their strong odor, which is an important factor in consumer formulations.

The two selected solvents were tested against the current solvent blend and toluene as a reference. As a first test, solubility of the neoprene rubber was tested by mixing it with the solvents in a 1:9 mass ratio. The mixtures were mixed at the roller bench for 24 h at 6 rpm. The rubber dissolved in all of the solvents. Visual differences in viscosity of the mixture were observed, and the viscosity increased in the order of solvent blend < toluene < *p*-cymene < isobutylbenzene.

Next, formulations were made based on the current formula. Viscosity was measured on the completed formulations with resin added. Viscosity was determined using a Brookfield RVDV-E apparatus at 20 rpm and at 23 °C/RH 50%, applying the appropriate spindle (Sp5). The viscosity for the finished formulations is approximately a factor 1.5 times higher compared to the toluene formulation and a factor 4–5 times higher compared to the current solvent blend (Table 3). The toluene formulation, however, also shows a viscosity 3 times higher compared to the solvent blend.

**Figure 12:**
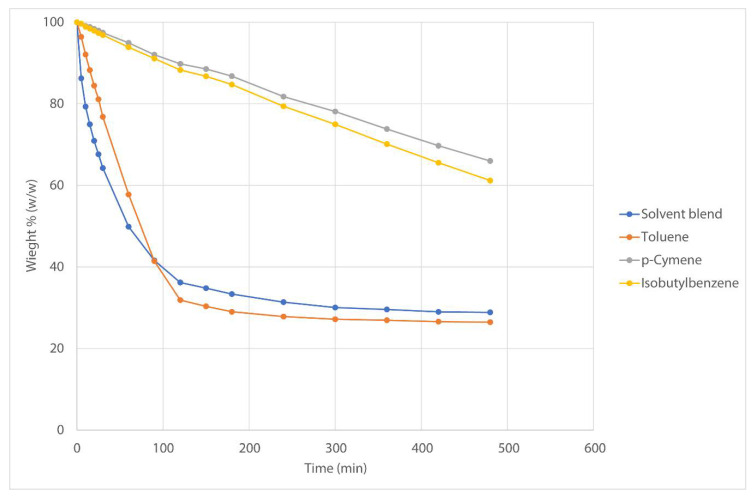
Evaporation rates for finished formulations for reference solvents and replacement solvents.

To test the evaporation rate, the formulations were placed in aluminum cups and air dried. The weight was recorded in function of time and plotted as weight percentage (Figure 12). The replacement solvents clearly show a much slower rate of evaporation than the solvent blend and toluene formulation. The solid base of the adhesive formulation is 22.5%. After 72 h, all formulations were completely dried. The drying times do not reflect a real-use situation since the adhesives were not applied and dried as a film.

At that time, a satisfactory one-one-one replacement could not be found. Price, odor, or evaporation speed were the limiting factors.

#### 2.2.3. Benchmark of TMO

The SUSSOL software can also be used as a benchmark for new solvents. For example, tetramethyl oxolane (TMO) or 2,2,5,5-tetramethyltetrahydrofuran (TMTHF) is presented as a renewable substitute for toluene amongst others [59]. The Hansen solubility parameters, however, are not very close to the values for toluene (Hansen distance of 5.3 MPa^1/2^), which makes the solvent a possible substitute for a range of other solvents. Using the clustering analysis in the software, similar (nonbenign) solvents can easily be detected. Not all physical properties in our dataset are available for TMO. The clustering is performed with a dataset containing 13 properties (instead of 22).

From the clustering, it can be concluded that TMO is a valid substitute for toluene and similar aromatic compounds, as published by Byrne et al. [59]. Toluene, benzene, and other alkyl substituted aromatic compounds, such as xylenes and propylbenzene, are significant neighbors of TMO. In Table 4, a selection of the candidates list is presented. All solvents within a distance of 6 MPa^1/2^ in the Hansen space that have a high score (>5) for safety, health, or environment are included. Amines were removed from the list because the use of amines is usually related to their basicity.

Common hydrocarbon solvents such as *n*-hexane and traditional ethers such as diethyl ether are also present in the candidates list generated by the software. Additionally, esters and ketones containing 5–8 carbon atoms are clustered together with TMO. More interestingly, some halogenated solvents are replaceable with TMO. For example, 1,1-Dichloroethene, 1,1-dichloroethane, 1,1,1-trichloroethane, and 1,1-dichloropropane are all significant neighbors of TMO. All of these solvents score very high on health and/or environmental impact. In addition, ethylene glycol diethyl ether (1,2-diethoxyethane), which scores a 9 for health could be substituted with TMO. Certainly, the substitution should be tested for each case individually, depending on the application, however, using the software like this supports the search for possible applications where TMO could be a successful substitute and accordingly, could aid solvent producers and distributors.

## 3. Materials and Methods

In the following section, the software is described in detail. Algorithms and underlying principles are discussed comprehensively. A version of the software with limited functionality and a small dataset is available on GitHub [48]. The implementation of the SOM algorithm in Java can be found in a GitHub repository [60] and the MDS algorithm used in SUSSOL is available at the website of the University of Konstanz [61].

### 3.1. Sustainable Solvents

A lot has been published on “how green is a solvent” [34]. Furthermore, every industry or company has its own priorities. Therefore, it was decided to integrate the CHEM21 approach [33] in our software. This approach is robust since it requires less data, and it is a recognized methodology. The CHEM21 methodology assigns scores for safety, health, and environment criteria, aligned with the Global Harmonized System (GHS) and European regulations. Every criterion (SH&E) is assigned a score between 1 and 10, 10 representing the highest hazard in each category. A color code is associated with the scores: green for 1–3, yellow for 4–6, and red for 7–10. The combination of the three scores determines the final ranking of the solvent: hazardous, problematic, or recommended.

The safety score is assigned based on the flash point and boiling point. Penalty points are assigned for a low autoignition temperature, a high resistivity, or the ability to form peroxides. A solvent with a high energy of decomposition has a score of 10.

The health score is determined by the compounds H3xx statements. More severe H3xx statements yield higher scores. A penalty point is added to the score for low boiling point (<85 °C) solvents. Solvents with incomplete toxicological data are assigned a score of 5.

The score for environmental impact is based on the solvent’s boiling point, the H4xx statements, and the REACH status. An ideal boiling range between 70 and 139 °C is proposed. Solvents with lower boiling points are more likely to give rise to emissions, but a high boiling solvent is more difficult to recycle. Solvents with incomplete environment toxicity data are assigned a score of 5. Solvents with the H420 statement (ozone layer hazard) have a score of 10 by default.

The scores for SH&E and the corresponding color codes are self-explanatory and provide the user of our software with a reliable starting point to decide which solvents to further look into. Whenever the user selects a solvent in the graphical user interface (GUI), the final ranking and the scores for SH&E are shown. It is up to the user to base their decision on comprehensive data on toxicity, regulatory issues, cost, and availability and priorities within the industry. The only drawback in using the CHEM21 methodology [33] in the general-purpose solvent guide subject of this publication is its clear focus on pharmaceutical companies. The importance of the boiling point in the score for environmental impact is inconvenient for applications that do not require the solvent to be separated via distillation (e.g., paints, coatings, and cleaning agents).

### 3.2. Dataset

Common organic solvents were included together with, “green,” neoteric, and biobased solvents. A solvent distributor and several solvent users (a paint and coatings producer, a producer of industrial cleaning agents, a pharmaceutical company, and a fine chemicals company) were asked which solvents should be incorporated in the dataset. This produced a list of about 200 “common” organic solvents which was complemented with “new” and alternative solvents. Gasses, supercritical fluids, ionic liquids, and deep eutectic solvents were excluded from the dataset.

**Figure 13:**
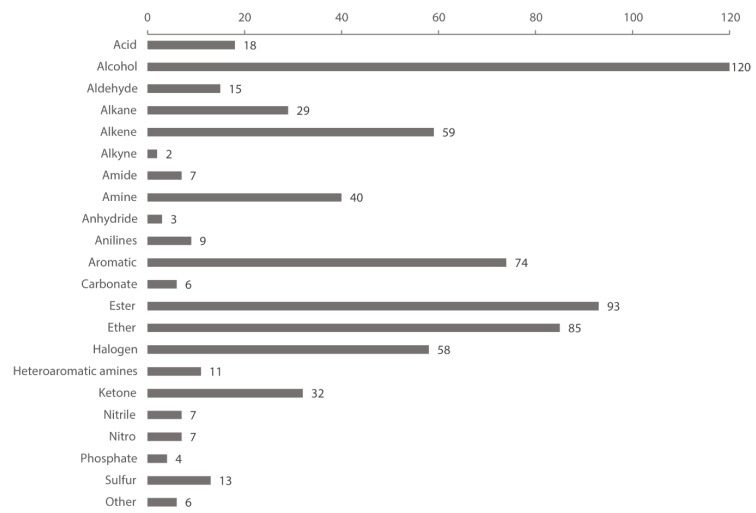
Functional groups in the dataset.

The present dataset contains 500 solvents. A list of the compounds in the dataset is available in the Supporting Information. An overview of functional groups present in the dataset is depicted in Figure 13. A solvent may belong to multiple classes, as some solvents contain multiple functional groups (e.g., diacetone alcohol). Alcohols, alkenes, esters, ethers, aromatic compounds, and halogenated compounds together account for 70% of the total functional group count (698).

Specifically, 25 Solvents in the dataset are solvents in the SVHC list and 54 solvents are in the SIN list (Table 5). Following the CHEM21 ranking of solvents with a green, amber, or red color, 349 solvents in the dataset have a green safety score, 276 solvents have a green health score, and only 65 solvents have a green environmental score. This is due to the importance of the boiling point in assigning the environment score. Solvents with a boiling point lower than 70 °C or above 139 °C are scored amber or red [33]. In total, 48 solvents have a boiling point below 70 °C and 303 solvents have a boiling point above 139 °C. Overall, 118 solvents in the dataset are ranked as “recommended” according to the CHEM21 methodology.

Every solvent in the dataset is described by 22 physical properties (Table 6). Sources used to compile the dataset include supplier and producer information (Sigma Aldrich [62], Acros Organics [63], Fisher Scientific [64], Merck Millipore [65], VWR [66], Huntsman [67], Dow [68], BASF [69], Shell [70], Solvay [71], and Total [72]), online databases (PubChem [73], ChemSpider [74], Chemicalbook [75], Molbase [76], and ECHA [77]), commercially available database programs (Chemtec [78] and HSPiP [79]), and handbooks (CRC Handbook of Chemistry and Physics [80] and Sustainable Solvents RSC Green Chemistry Series [81]).

The solvent dataset is loaded in the software in the form of a .csv file and thus provides the necessary flexibility to the user. Solvents (rows) can be added or removed as well as physical properties (columns). The .csv format allows to export/import solvent subsets to/from other software packages, such as HSPiP [79].

### 3.3. Solvent Selection

#### 3.3.1. Multidimensional Scaling (MDS) Plot

MDS uses a square symmetrical matrix of the Euclidean distances between all solvents, called the similarity matrix. This matrix is then compared to the original full dataset by evaluating a stress function. Stress is a goodness-of-fit measure, based on differences between predicted and actual distances [51,52]. The coordinates in 2D are then adjusted, if necessary, to reduce stress.

The stress function is defined as:

(2) $$\frac{\sum \sum (f(x_{ij})-d_{ij})²}{\mathrm{scale}}$$

In Equation (2), d_ij_ refers to the Euclidean distance, across all dimensions, between solvents i and j on the map, f(x_ij_) is a function of the input data, and scale refers to a constant scaling factor, used to keep stress values between 0 and 1. When the MDS map perfectly reproduces the input data, f(x_ij_) − d_ij_ is 0 for all i and j, so stress is zero. The smaller the stress, the better the representation or dimensionality reduction. The transformation of the input values f(x_ij_) depends on whether metric or non-metric scaling is used. In metric scaling, which applies to the solvent data set, f(x_ij_) = x_ij_.

For some datasets, it is easy to define the x and y axes of the resulting 2D map, e.g., for distances between cities. For high-dimensional datasets, the dimensions are abstracted, and that is why, the axes in our MDS plot are undefined.

#### 3.3.2. Filter Module

The user can explore the solvent dataset by selecting individual solvents in the MDS plot. In an associated filter module, it is possible to define ranges for the physical properties, thus obtaining a set of solvents that meet the user’s criteria. Criteria can be combined, resulting in even more fine-grained filters. This allows the user to conveniently create subsets of solvents with desirable properties for the user’s intended application.

### 3.4. Solvent Substitution

#### 3.4.1. Self-Organizing Map

For solvent substitution, we apply a clustering algorithm, the Self-organizing Map (SOM) of Kohonen [49,50]. The SOM is a neural network, based on neurophysiological research by Hubel and Wiesel [82], who established the existence of ocular dominance columns in the visual cortex of a cat. Ocular dominance columns are stripes of neurons in the visual cortex of certain mammals, including humans, that respond preferentially to input from one eye or the other and to lines with a specific orientation.

The SOM can be seen as an artificial implementation of this neuroanatomical structure, consisting of a comparable grid of artificial neurons. It has been used extensively as a visualization tool in exploratory data analysis. It has had plenty of practical applications ranging from industrial process control and finance analyses to the management of very large document collections.

Initially, we tested several other clustering algorithms (K-Means, Canopy, DBSCAN, COBWEB, and Expectancy Maximization) [83] on small datasets to be able to choose the most suited one, from a chemical viewpoint. The datasets were compiled in such a way that several obvious clusters were present in the data. For example, a dataset with 3 short-chain alcohols, 3 alkanes, and 3 ketones was used. Using these datasets, the resulting clusterings could easily be evaluated. The SOM algorithm produced the most chemically relevant clusterings.

**Figure 14:**
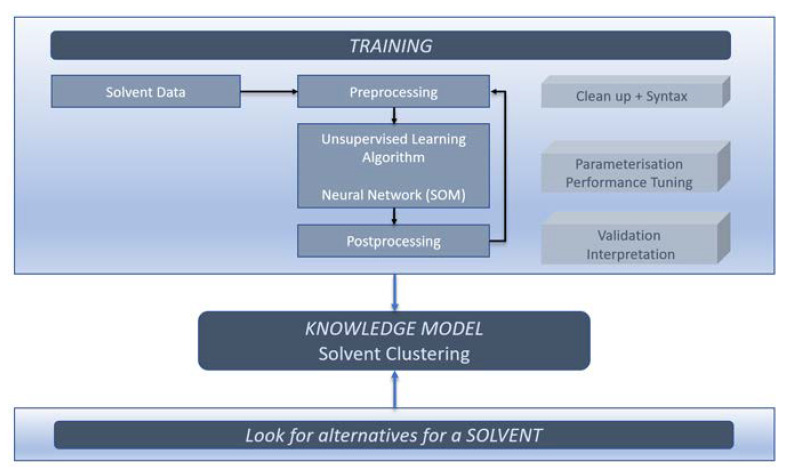
AI chain of events.

Regardless of the choice of algorithm, in AI learning, a specific procedure is followed, see Figure 14.

Solvent data has to be entered in the dataset and cleaned according to a specific syntax. The solvent data is then offered to the algorithm for clustering. Data for clustering only include the physical properties (numeric) data from the dataset. The SH&E scores are not used for clustering but are assigned to the solvent succeeding the clustering. The resulting model must be validated both chemically and statistically. If the model is not satisfactory, the cycle repeats. The dataset can be adjusted to contain more or less solvents or to give more or less weight to specific physical properties.

Three hyperparameters highly influence the behavior of the SOM: learning rate, width, and height. The learning rate can be adjusted to change how coarse learning proceeds. By changing the width and height of the map, the total number of neurons is increased or decreased. When finally the model has converged, it can be used to look for alternative, comparable solvents.

During training, all solvents are offered to the SOM grid and allocated to a specific column or neuron. Comparable solvents are grouped together around 1 specific neuron, which results in clustering. In pseudocode, this looks like:
**Input**: a set of solvents, *X* = {*x*_1_, ..., *x*_n_}**Output**: a set of clusters, *Y* = {*y*_1_, ..., *y*_n_}**Begin**  Initialize *Y* = {*y*_1_, ..., *y*_n_} randomly  **Repeat** select *x* ∈ *X* randomly    Find y^*^ such that d(x,y^*^) = min{d(x,y)| y ∈ Y}    **For** all y ∈ N(y^*^)
**do**      *y = y + γ (x− y)*    Reduce learning rate *γ*  **Until** termination condition is true**End**

N(y*) = the neighborhood of y*

γ = the learning rate which determines how coarse learning proceeds

#### 3.4.2. Statistical Postprocessing

Results of neural network learning are always subject to some variability due to the sensitivity to initial conditions, to convergence to local minima, and, sometimes more dramatically, to sampling variability [84]. Consequently, the content of clusters (groups of solvents that are similar) may vary for each run. To illustrate this, 250 runs were performed on a small solvent set (57 solvents) containing all solvents in the 500 solvents dataset within a distance of 4 MPa^1/2^ (according to the Seymour rule of thumb [58]) from d-Limonene in the Hansen solvent space. In Table 7, the solvents present in the same cluster as d-Limonene are listed for the first 10 runs.

The results may vary for each run, but it is clear that some solvents are more often in the “limonene cluster” than others. Decalin is 6 out of 10 times present in the same cluster as limonene, whereas other solvents are clustered only once together with limonene. If the clustering analysis is performed a sufficient number of times, it is possible to do a statistical postprocessing analysis and determine which solvent pairs are statistically significant clustered in the same cluster. Further, it is explained what “a sufficient number of times” might be.

To implement this assessment, de Bodt et al. [84] introduced the objective measure of neighborhood.

(3) $$\mathrm{NEIG}H_{i,\;j}^{b}\left(r\right)=\left\{\begin{array}{c} 0\;\mathrm{if}\;x^{i}\;\mathrm{and}\;x^{j}\;\mathrm{are}\;\mathrm{not}\;\mathrm{neighbor}\;\mathrm{within}\;\mathrm{radius}\;r\\ 1\;\mathrm{if}\;x^{i}\;\mathrm{and}\;x^{j}\;\mathrm{are}\;\mathrm{neighbor}\;\mathrm{within}\;\mathrm{radius}\;r \end{array}\right\}$$

Being neighbors within radius r means that two solvents x^i^ and x^j^ are within the same cluster or in neighboring clusters within the radius. If the radius r is 0 and NEIGH_i,j_ = 1, the two solvents are projected in the same cluster. If r > 0, also surrounding clusters are taken into account. In this work, we adopt r = 0 for all assessments. Superscript b in Equation (3) signifies that the result for the NEIGH_i,j_ value is obtained for a specific clustering (simulation or run) b; of course, it may happen that two solvents x^i^ and x^j^ are neighbors after one simulation and not after another as illustrated in Table 7; this is exactly what we are looking for to evaluate. 

As a result of Equation (3), the neighborhood value NEIGH_i,j_ for the solvent pair x^i^, x^j^ is 0 if they are not in the same cluster and 1 if they are.

Next, a stability function (STAB_i,j_) is defined [84] as the average of NEIGH_i,j_ over all simulations

(4) $$\mathrm{STA}B_{i,j}\left(r\right)=\frac{\sum_{b=0}^{B}\mathrm{NEIG}H_{i,j}^{b}\left(r\right)}{B},$$

where B is the total number of simulations.

The STAB_i,j_ function is a measure to evaluate if it is meaningful that a solvent pair x^i^, x^j^ is clustered within the same cluster. To assess this, we check if NEIGH_i,j_ always has the same value (0 or 1) over all simulations. A perfect stability would lead STAB_i,j_ to be 0 (x^i^ and x^j^ are never neighbors) or 1 (they are always neighbors).

In practice, STAB_i,j_ is a value between 0 and 1. The higher the STAB_i,j_ value, the more certain we are that solvents x^i^ and x^j^ belong to the same cluster and thus, exhibit similar physical properties. As an example, a table with STAB values for the previous clustering of limonene is included (Table 8). In 36% of the runs, α-pinene is present in the same cluster as limonene. α-Pinene is a C10 terpene just like limonene, hence it is the solvent which has the highest neighbor count in the dataset after 250 runs. Cyclic hydrocarbons (mostly substituted cyclohexanes) also appear to be highly similar to limonene. In addition, some aromatic solvents seem quite alike. *o*-Diethylbenzene is clustered together with limonene 48 out of 250 times.

Finally, it is possible to study the significance of the neighborhood for a specific pair of solvents by comparing STAB_i,j_ to the value it would have if the solvents fell in the same cluster in a completely random way (unorganized map). If this specific pair of solvents only falls in the same cluster by chance, then the STAB_i,j_ statistic will be approximately equal to the value it would take in unorganized maps. Comparing the STAB_i,j_ values in these two situations (organized and unorganized maps) is thus a way to make possible the use of a conventional statistical test to check if the STAB_i,j_ statistic in the organized case is significant or not. If U is the total number of possible clusters (which corresponds to the total number of centroids in the SOM) and ν is the size of the neighborhood (which can be calculated from the radius r by $\nu ={\left(2r+1\right)}^{2}$, but is always 1 when r = 0), the probability of x^i^ and x^j^ being neighbor in the random case is ν/U.

With the probability of success being ν/U (in this case, success means x^i^ and x^j^ are neighbors), the number Y of successes in B simulations is distributed as a binomial distribution, with parameters B and ν/U. This allows to test the H_0_ hypothesis: x^i^ and x^j^ are random neighbors against H_1_: the fact that x^i^ and x^j^ are (not) neighbors is meaningful. An approximated Gaussian distribution can be used if the conditions Bν/U > 10 and B(1 – ν/U) > 10 are met. Since we use r = 0 (ν = 1), this implies that the number of runs should be at least a factor of 10 higher than the number of centroids in the SOM. Consequently, if Y is less than $B\;\frac{\nu }{U}-1.96\;\sqrt{B\;\frac{\nu }{U}\left(1-\frac{\nu }{U}\right)}$ or greater than $\;B\;\frac{\nu }{U}+1.96\;\sqrt{B\;\frac{\nu }{U}\left(1-\frac{\nu }{U}\right)}\;$, H_0_ is rejected, and we can conclude with a confidence level of 5% that the fact that x^i^ and x^j^ are (not) neighbors is meaningful. If the STAB_i,j_ value is close to 0, x^i^ and x^j^ are significant not neighbors and if STAB_i,j_ is close to 1, x^i^ and x^j^ are significant neighbors. For the analysis in Table 8, 25 centroids were used in the SOM and 250 runs were performed. The upper and lower limit are thus 3.9 and 16.1. Consequently, all solvents with a neighbor count higher than 16 are statistically neighbors of limonene.

This statistical analysis is automated in the software and serves as a postprocessing module. When searching for an alternative for solvent x, the significance of all solvent pairs with solvent x is checked. In this way, we generate a candidate list with all statistically significant substitution candidates for solvent x. The candidate list for d-Limonene is showed in Table 8. From this list, it can be concluded that limonene is a good substitute for longer chain hydrocarbon solvents (C9–C16) and alkylbenzenes, such as isopropylbenzene or the Shellsol A100 solvent, which is a C9–C10 aromatic hydrocarbon solvent.

## 4. Conclusions

All-purpose, fully automated, and AI based solvent selection and substitution software has been developed in this study. The SUSSOL software makes intuitive sense, and in most case studies, the software confirms the findings in literature, thus providing a sound platform for selecting the most sustainable solvent candidate. SUSSOL has also been tested in a real-life industrially relevant problem. The clustering and consecutive statistical analysis in software provide an easy-to-use approach, which is tunable for individual applications. Due to the extensive dataset and clustering principle, SUSSOL can serve as a starting point and a source of inspiration in the search for alternative solvents, especially to nonexperts. The software is complementary with other software packages such as HSPiP, since it is easy to make subsets and export them in the .csv format. The dataset is easily extendable with other physical properties and/or solvents.

## Figures and Tables

**Table 1:** Short list for NMP (*N*-methyl pyrrolidone)replacement.

Cas Number	Name	S Score	H Score	E Score	Overall Ranking
67-68-5	Dimethyl sulfoxide	1	1	5	Problematic
53716-82-8	Cyrene	1	2	7	Problematic
3470-98-2	*N*-Butyl pyrrolidone	1	2	7	Problematic
110-86-1	Pyridine	3	2	3	Hazardous ^a^
67-64-1	Acetone	5	3	5	Problematic
105-45-3	Methyl acetoacetate	1	4	5	Problematic
872-50-4	*N*-Methyl pyrrolidone	1	9	7	Hazardous
2687-91-4	1-Ethyl pyrrolidone	1	9	7	Hazardous
127-19-5	Dimethylacetamide	1	9	5	Hazardous
68-12-2	Dimethylformamide	3	9	5	Hazardous
54-11-5	Nicotine	1	9	7	Hazardous

^a^ The default scores as determined by the CHEM21 methodology do not reflect the low occupational limit values of pyridine, therefore, the overall ranking was changed to “hazardous” by the CHEM21 solvent team.

**Table 2:** Short list with solvent replacement candidates for toluene in a contact adhesive.

Cas Number	Name	S Score	H Score	E Score	Overall Ranking
538-93-2	Isobutylbenzene	3	2	5 ^a^	Recommended
99-87-6	*p*-Cymene	4	2 ^b^	5 ^b^	Problematic
135-01-3	*o*-Diethylbenzene	3	2	7	Problematic
103-65-1	Propylbenzene	3	2	7	Problematic
138-86-3	Dipentene	4	2	7	Problematic
5989-27-5	R-limonene	4	2	7	Problematic
110-83-8	Cyclohexene	5	3	7	Problematic
105-05-5	1,4-Diethylbenzene	3	4	7	Problematic
80-56-8	α-Pinene	3	4	7	Problematic
108-88-3	Toluene	5	6	3	Problematic
100-40-3	4-Vinylcyclohexene	5	6	5	Problematic
119-64-2	1,2,3,4-Tetrahydronaphthalene	2	6	7	Problematic
95-49-8	*o*-Chlorotoluene	3	6	7	Problematic
101-83-7	Dicyclohexylamine	1	7	7	Hazardous
98-51-1	4-tert-Butyltoluene	3	9	5	Hazardous
98-07-7	Benzotrichloride	1	9	7	Hazardous

^a^ Isobutylbenzene is not fully REACH-registered and did not show the H410 statement at the time of testing. At the time of writing, isobutylbenzene scores a 7 on environment. As a result, the overall ranking changes to “problematic.” ^b^ At the time of testing, p-Cymene was REACH-registered for intermediate use only and showed H226, H315, H319, and H304 statements. Meanwhile, the substance is fully registered with H361 and H411 statements. As a result, the scores for health and environment change to 6 and 7, respectively. The overall ranking remains valid. This illustrates the importance of an up-to-date dataset and the necessity to review each solvent replacement candidate thoroughly before proceeding.

**Table 3:** Viscosity for solvent rubber mixture and finished formulations for the reference solvents and the replacement solvents.

Viscosity (mPa.s)	Solvent Blend	Toluene	*p*-Cymene	Isobutylbenzene
Finished formulation	3300	9900	17,000	13,700

**Table 4:** Short list with selected solvents from candidates list for tetramethyl oxolane (TMO) benchmark.

Cas Number	Name	S Score	H Score	E Score	Overall Ranking
75-35-4	1,1-Dichloroethene	8	10	7	Hazardous
110-00-9	Furan ^a,b^	8	10	7	Hazardous
96-05-9	Allyl methacrylate	3	9	7	Hazardous
629-14-1	Ethylene glycol diethyl ether ^a,b^	5	9	5	Hazardous
107-83-5	2-Methyl pentane	7	7	7	Hazardous
110-54-3	Hexane ^a^	5	7	7	Hazardous
75-34-3	1,1-Dichloroethane	5	7	7	Hazardous
542-92-7	Cyclopentadiene	3	7	7	Hazardous
106-88-7	1-Butylene oxide	7	7	5	Hazardous
96-47-9	2-Methyl tetrahydrofuran	5	7	3	Problematic
1569-69-3	Cyclohexanethiol	3	6	7	Problematic
100-40-3	4-Vinylcyclohexene	5	6	5	Problematic
626-38-0	sec-Amyl acetate	5	6	5	Problematic
431-03-8	2,3-Butanedione	5	6	5	Problematic
78-99-9	1,1-Dichloropropane	5	6	5	Problematic
140-88-5	Ethyl acrylate	5	6	5	Problematic
591-78-6	2-Hexanone	4	6	5	Problematic
123-05-7	2-Ethylhexanal	4	6	5	Problematic
628-63-7	*n*-Amyl acetate	3	6	5	Problematic
108-88-3	Toluene	5	6	3	Problematic
71-55-6	1,1,1-Trichloroethane ^a^	4	3	10	Hazardous
76-13-1	1,1,2-trichlorotrifluoroethane	1	3	10	Hazardous
60-29-7	Diethyl ether	10	3	7	Hazardous

^a^ Solvent present in ChemSec’s SIN-list and ^b^ solvent present in REACH SVHC-list.

**Table 5:** Solvents in the dataset mentioned in Substitute It Now (SIN)-list and/or substances of very high concern (SVHC)-list.

CAS Number	Solvent	CAS Number	Solvent
71-55-6	1,1,1-Trichloroethane ^a^	111-15-9	2-Ethoxyethyl acetate ^a,b^
112-49-2	1,2-Bis(2-methoxyethoxy)ethane ^a,b^	629-14-1	1,2-Diethoxyethane ^a,b^
95-50-1	1,2-Dichlorobenzene ^a^	110-49-6	2-Methoxyethyl acetate ^a,b^
123-91-1	1,4-Dioxane ^a^	75-12-7	Formamide ^a,b^
106-89-8	1-Chloro-2,3-epoxypropane ^a^	110-00-9	Furan ^a,b^
2687-91-4	*N*-Ethyl-2-pyrrolidone ^a^	110-54-3	Hexane ^a^
107-13-1	Acrylonitrile ^a^	302-01-2	Hydrazine ^a^
62-53-3	Aniline ^a^	75-26-3	2-Bromopropane ^a^
71-43-2	Benzene ^a^	109-86-4	2-Methoxyethanol ^a,b^
119-61-9	Benzophenone ^a^	79-16-3	*N*-methylacetamide ^a,b^
98-07-7	Benzotrichloride ^a^	123-39-7	*N*-methylformamide ^a^
100-44-7	Benzyl chloride ^a^	1634-04-4	Tert-butyl methyl ether ^a^
75-15-0	Carbon disulfide ^a^	91-20-3	Naphthalene ^a^
56-23-5	Carbon tetrachloride ^a^	98-95-3	Nitrobenzene ^a,b^
106-47-8	4-Chloroaniline ^a^	79-46-9	2-Nitropropane ^a^
67-66-3	Chloroform ^a^	872-50-4	*N*-methyl-2-pyrrolidone ^a,b^
107-15-3	Ethylenediamine ^a,b^	106-94-5	1-Bromopropane ^a,b^
84-74-2	Dibutyl phthalate ^a,b^	75-56-9	Propylene oxide ^a,b^
107-06-2	1,2-Dichloroethane ^a,b^	91-22-5	Quinoline ^a^
78-87-5	1,2-Dichloropropane ^a^	100-42-5	Styrene ^a^
84-66-2	Diethyl phthalate ^a^	96-09-3	Styrene oxide ^a^
64-67-5	Diethyl sulfate ^a,b^	97-99-4	Tetrahydrofurfuryl alcohol ^a^
111-96-6	Diglyme ^a,b^	95-53-4	*o*-Toluidine ^a,b^
110-71-4	1,2-Dimethoxyethane ^a,b^	87-61-6	1,2,3-Trichlorobenzene ^a^
77-78-1	Dimethyl sulfate ^a,b^	120-82-1	1,2,4-Trichlorobenzene ^a^
127-19-5	*N*,*N*-dimethylacetamide ^a,b^	79-01-6	Trichloroethylene ^a,b^
110-80-5	2-Ethoxyethanol ^a,b^	96-18-4	1,2,3-Trichloropropane ^a,b^

^a^ Solvent present in ChemSec’s SIN-list and ^b^ solvent present in REACH SVHC-list.

**Table 6:** Physical properties in the dataset with associated units.

Property	Units
Boiling point	°C
Melting point	°C
Vapor pressure	mmHg (at 25 °C)
Flash point	°C
Autoignition temperature	°C
Hansen delta D	MPa^1/2^
Hansen delta P	MPa^1/2^
Hansen delta H	MPa^1/2^
Solubility in water	g/L (at 20 °C)
Density	kg/L (at 25 °C)
Viscosity	mPa.s (at 25 °C)
Relative vapor density	(Air = 1)
Log P octanol water	(at 20 °C)
Refractive index	(at 20 °C)
Surface tension	mN/m (at 25 °C)
Molar volume	mL/mol
Relative evaporation rate	(BuAc = 100)
Antoine A	/
Antoine B	/
Antoine C	/
Molecular weight	g/mol
Log S	(at 20 °C)

**Table 7:** Contents of “limonene cluster” for first 10 runs.

Solvent	Run Number										Count
	1	2	3	4	5	6	7	8	9	10	
*cis*-Decalin	x		x			x	x	x	x		6
α-Pinene	x		x		x	x			x		5
Dihexyl ether	x		x		x		x	x			5
Butylcyclohexane		x	x			x	x				4
Undecane	x			x			x				3
1-Decene		x		x	x						3
Tridecane	x						x	x			3
Propylcyclohexane		x	x								2
Tributylamine		x	x								2
1-Tetradecene	x						x				2
Pentadecane	x						x				2
Hexadecane	x						x				2
Tetradecane	x						x				2
Orange terpene	x										1
*o*-Diethylbenzene									x		1
1,2,4-Trimethylbenzene			x								1
Dodecane	x										1
Decane				x							1
Varsol 60				x							1
*p*-Diethylbenzene										x	1
1,3-Dimethylcyclohexane										x	1

**Table 8:** Candidate list for limonene. Neighbor counts after 10 and 250 runs and STAB (stability) values.

Solvent	Count after 10 Runs	Count after 250 Runs	STAB Value
α-Pinene	5	90	0.36
Butylcyclohexane	4	78	0.31
*cis*-Decalin	6	74	0.30
Propylcyclohexane	2	67	0.27
Orange terpene	1	53	0.21
Tributylamine	2	51	0.20
Dihexyl ether	5	49	0.20
*o*-Diethylbenzene	1	48	0.19
Isopropylbenzene	0	47	0.19
1-Decene	3	39	0.16
Undecane	3	39	0.16
Ethylbenzene	0	34	0.14
1-Tetradecene	2	28	0.11
Pentadecane	2	24	0.10
Tridecane	3	23	0.09
Shellsol A100	0	22	0.09
Hexadecane	2	21	0.08
1,2,4-Trimethylbenzene	1	21	0.08
Dodecane	1	20	0.08
Tetradecane	2	20	0.08

## Supplementary Materials

The following are available online at https://github.com/SUSSOLKDG/Sussol. A version of the SUSSOL software with limited functionality and a dataset containing 45 solvents is available. A list of the compounds in the dataset is available in the supporting information.
